# Severe Immune Thrombocytopenic Purpura in a Pediatric Patient With Fanconi Anemia: A Case Report

**DOI:** 10.7759/cureus.68973

**Published:** 2024-09-09

**Authors:** Lujain Alamri

**Affiliations:** 1 General Practice, King Khalid University, Abha, SAU

**Keywords:** autoimmune, bone marrow failure, fanconi anemia, immune thrombocytopenic purpura, pediatric, rituximab

## Abstract

Fanconi anemia (FA) is a rare inherited disorder characterized by progressive bone marrow failure, chromosomal instability, and an increased predisposition to malignancies. Autoimmune manifestations are uncommon in FA, with immune thrombocytopenic purpura (ITP) being a particularly rare presentation. ITP is an autoimmune disorder marked by immune-mediated platelet destruction, leading to severe thrombocytopenia and an increased risk of bleeding. This case report presents a pediatric patient with FA and severe ITP, illustrating the clinical challenges of managing autoimmune complications in the context of FA. This case report describes the case of a six-year-old boy with known FA who presented with a three-day history of spontaneous bruising, petechiae, and epistaxis. He had severe thrombocytopenia (platelet count: 8,000/µL) without other significant cytopenias. The initial workup excluded viral infections and other secondary causes, leading to the diagnosis of ITP. The patient was treated with intravenous immunoglobulin (IVIG) and corticosteroids, resulting in a transient improvement. However, his platelet counts declined, prompting treatment with rituximab, which achieved a sustained response. He was discharged after four weeks of rituximab therapy and remained stable at follow-up with a platelet count of 100,000/µL. This case highlights the rare occurrence of ITP in FA and the successful use of rituximab for refractory thrombocytopenia. The findings suggest a need for ongoing research into the mechanisms of immune dysregulation in FA and the development of optimized therapeutic strategies for managing autoimmune manifestations in this complex patient population.

## Introduction

Fanconi anemia (FA) is a rare, inherited disorder characterized by chromosomal instability, progressive bone marrow failure, and an increased predisposition to malignancies. It is the most common inherited form of aplastic anemia, typically diagnosed in childhood. The disease is caused by mutations in genes responsible for DNA repair, leading to genomic instability and pancytopenia [1,2]. Patients with FA frequently present with physical abnormalities such as short stature, abnormal skin pigmentation, and congenital malformations, although the severity and range of manifestations vary. Hematological complications, particularly bone marrow failure, are the hallmark of the disease and can progress to myelodysplastic syndromes or acute myeloid leukemia [1-3].

Autoimmune manifestations in FA are rare but have been increasingly reported, with immune thrombocytopenic purpura (ITP) being one such uncommon presentation. ITP is an autoimmune disorder characterized by immune-mediated platelet destruction, leading to severe thrombocytopenia and an increased risk of bleeding. While ITP can occur idiopathically or in association with other autoimmune conditions, its occurrence in the context of FA is particularly unusual [2-4].

## Case presentation

A six-year-old boy with a known history of FA presented to the emergency department with a three-day history of spontaneous bruising and petechiae, primarily on the extremities, and episodes of epistaxis. His parents denied any history of recent trauma or bleeding, and there was no significant family history of bleeding disorders. The patient was previously diagnosed with FA at age four after presenting with short stature and abnormal skin pigmentation and had since been under hematologic follow-up. His clinical course had been characterized by bone marrow failure, with occasional episodes of mild pancytopenia, but he had not required any blood product transfusions or developed any severe infections.

On arrival, the patient appeared pale but was hemodynamically stable, with no signs of acute distress. His vital signs were within normal limits, with a heart rate of 98 beats per minute, a respiratory rate of 20 breaths per minute, a blood pressure of 98/60 mmHg, and an oxygen saturation of 98% on room air. Physical examination revealed multiple ecchymoses and petechiae on his arms, legs, and torso but no signs of active bleeding from mucosal surfaces. There were no signs of hepatosplenomegaly or lymphadenopathy, and the neurological exam was unremarkable. No other significant abnormalities were noted.

Initial laboratory investigations demonstrated severe thrombocytopenia, with a platelet count of 8,000/µL (reference range: 150,000-450,000/µL), hemoglobin of 10.2 g/dL (reference range: 11.5-15.5 g/dL), and a white blood cell count of 3,200/µL (reference range: 5,000-14,500/µL). The reticulocyte count was low, consistent with his history of FA-related bone marrow failure. His coagulation profile, including prothrombin time (PT), activated partial thromboplastin time (aPTT), and fibrinogen levels, were all within normal limits. A peripheral blood smear revealed marked thrombocytopenia with no significant schistocytes or atypical cells. Bone marrow aspiration and biopsy were deferred at this stage due to the patient's severely low platelet count.

Given his profound thrombocytopenia in the context of FA, a differential diagnosis including bone marrow failure-related cytopenias, viral infections (such as Epstein-Barr virus and cytomegalovirus), drug-induced thrombocytopenia, and immune-mediated destruction (such as ITP) was considered. Viral serologies for Epstein-Barr virus, cytomegalovirus, and parvovirus B19 were negative. Inflammatory markers, including C-reactive protein and erythrocyte sedimentation rate, were normal. An autoimmune screen was negative, including antinuclear antibodies (ANA) and anti-double-stranded DNA (dsDNA). No recent changes in medication or exposure to new drugs were reported. Given these findings, the most likely diagnosis was ITP as an autoimmune manifestation in the setting of underlying FA.

Management was initiated with intravenous immunoglobulin (IVIG) at a dose of 1 g/kg/day for two days, along with corticosteroids (prednisolone 2 mg/kg/day) to address the suspected autoimmune etiology of thrombocytopenia. Due to the severity of his thrombocytopenia and risk of bleeding, he was closely monitored in the pediatric intensive care unit. Platelet transfusions were administered intermittently to prevent spontaneous hemorrhage, although it was acknowledged that transfused platelets would likely be rapidly destroyed due to the underlying immune process.

Over the course of the next 72 hours, the patient showed a transient improvement in platelet counts, which rose to 22,000/µL, but continued to experience significant bruising and epistaxis. As a result, a second round of IVIG was administered, and the corticosteroid dose was gradually tapered. Despite initial improvement, his platelet counts again began to decline, leading to the consideration of additional therapies such as rituximab or thrombopoietin receptor agonists (TPO-RAs). After multidisciplinary discussions involving hematology, immunology, and pediatric teams, it was decided to initiate rituximab at a dose of 375 mg/m^2^ weekly for four weeks, given the refractory nature of his thrombocytopenia.

The patient tolerated rituximab without significant adverse effects and began to demonstrate a sustained increase in platelet counts by the third week of therapy, eventually reaching 75,000/µL by the fourth week. Steroids were further tapered, and the patient was discharged home on close outpatient follow-up with a plan for continued monitoring of his platelet levels and assessment of any potential long-term autoimmune complications.

At follow-up one month after discharge, the patient remained in stable condition, with a platelet count of 100,000/µL. He had no further episodes of bleeding or bruising, and his corticosteroids were fully tapered off. Given his underlying FA, which predisposes him to both bone marrow failure and immune dysregulation, ongoing monitoring for potential late effects, such as relapse of ITP or development of other autoimmune conditions, was planned. Genetic counseling and continued surveillance for hematologic and non-hematologic complications related to FA were also recommended.

## Discussion

The coexistence of FA and ITP in pediatric patients is rare, and the precise mechanisms underlying this association remain unclear. FA is a genetic disorder characterized by defective DNA repair pathways, leading to increased chromosomal fragility and progressive bone marrow failure. It predisposes affected individuals to hematologic complications such as aplastic anemia, myelodysplastic syndrome, and leukemia, but autoimmune manifestations are less commonly documented. Among the autoimmune disorders reported in FA, ITP, a condition driven by immune-mediated platelet destruction, represents an unusual and underrecognized entity, necessitating a thorough exploration of the pathophysiology and treatment strategies [2-4].

The relationship between FA and the development of autoimmune conditions such as ITP may be multifactorial. FA is associated with intrinsic bone marrow abnormalities and immune dysregulation due to defective DNA repair. The immunological dysregulation observed in FA likely involves a combination of impaired lymphocyte function, chronic inflammation, and a predisposition to autoantibody production, leading to immune-mediated cytopenias. Some studies have suggested that abnormal T-cell regulation, cytokine production, and B-cell dysregulation contribute to autoimmunity in FA patients. The inability of FA cells to adequately repair DNA damage may also provoke immune-mediated responses against hematopoietic cells, triggering conditions such as ITP [3,5].

In this case, the patient presented with severe thrombocytopenia, and after an exhaustive workup ruling out secondary causes, the diagnosis of ITP was made. While ITP is often idiopathic in children, the concurrence of FA complicates the diagnostic process and management. The absence of other viral or autoimmune triggers, alongside the known propensity for immune system abnormalities in FA, supports the hypothesis that ITP in this patient was a manifestation of immune dysregulation related to his underlying FA [2-4]. The identification of such cases is important, as it may guide future research to better understand the immunologic landscape in FA.

Treatment of ITP in patients with FA presents unique challenges. Standard first-line therapies for ITP, such as corticosteroids and IVIG, were initially employed in this case. Although the patient exhibited a partial response, the relapse and subsequent decline in platelet count after the initial treatment course highlight the refractory nature of ITP in this context. The use of immunosuppressive agents such as rituximab was necessary to achieve sustained remission [5-8]. This case reinforces the need for early recognition of refractory ITP in FA and the potential benefit of second-line therapies, including monoclonal antibodies such as rituximab, which targets B cells and mitigates autoantibody production.

However, the administration of immunosuppressive therapies in patients with FA requires careful consideration due to the underlying predisposition to infections and the risk of exacerbating bone marrow failure. While effective in treating ITP, rituximab poses potential risks, including prolonged B-cell depletion and increased susceptibility to infections [1-3]. In this case, the patient tolerated rituximab well, and no significant infectious complications occurred during follow-up. Nonetheless, close monitoring and a multidisciplinary approach are critical in balancing the benefits of immunosuppression against the potential risks in these complex patients.

Another therapeutic option for refractory ITP is TPO-RAs, which stimulate platelet production by promoting megakaryocyte maturation. While this class of drugs has shown promise in non-FA-related ITP, its utility in FA is less well established, given the underlying bone marrow pathology. The concern is that TPO-RAs could accelerate clonal evolution or leukemic transformation in FA, a well-known risk in these patients [2,4]. As such, their use should be considered cautiously, and further studies are needed to assess their safety and efficacy in the FA population.

This case also underscores the importance of long-term follow-up for FA patients, not only for the management of hematologic complications but also for the surveillance of autoimmune phenomena. The risk of recurrence of ITP or the development of other autoimmune conditions remains, particularly as FA is associated with chronic immune dysregulation. Continued monitoring is essential to assess for late effects of both FA and the therapies used to treat immune-mediated complications. Furthermore, genetic counseling is a crucial aspect of patient management, as FA is an inherited disorder with implications for family members.

## Conclusions

This case highlights the rare occurrence of severe ITP in a pediatric patient with FA, emphasizing the complexity of managing autoimmune manifestations in the context of an underlying bone marrow failure syndrome. The pathophysiological interplay between FA-related immune dysregulation and autoimmune cytopenias, such as ITP, remains incompletely understood but suggests a role for defective immune regulation and DNA repair. Successful management of refractory ITP in this patient using rituximab underscores the importance of individualized, multidisciplinary approaches in FA, balancing immunosuppressive therapy with the risks of infection and further hematologic complications. This case contributes valuable insights into the clinical challenges posed by FA-associated autoimmune disorders and highlights the need for ongoing research to optimize treatment and improve long-term outcomes for affected patients.
